# Application of compound material alleviates saline and alkaline stress in cotton leaves through regulation of the transcriptome

**DOI:** 10.1186/s12870-020-02649-0

**Published:** 2020-10-08

**Authors:** Mengjie An, Xiaoli Wang, Doudou Chang, Shuai Wang, Dashuang Hong, Hua Fan, Kaiyong Wang

**Affiliations:** Agricultural College, Shihezi University, Shihezi, Xinjiang 832000 People’s Republic of China

**Keywords:** Alkalinization, Antioxidant, Compound material, K^+^/Na^+^ ratio, Lignin biosynthesis, Salinization

## Abstract

**Background:**

Soil salinization and alkalinization are the main factors that affect the agricultural productivity. Evaluating the persistence of the compound material applied in field soils is an important part of the regulation of the responses of cotton to saline and alkaline stresses.

**Result:**

To determine the molecular effects of compound material on the cotton’s responses to saline stress and alkaline stress, cotton was planted in the salinized soil (NaCl 8 g kg^− 1^) and alkalized soil (Na_2_CO_3_ 8 g kg^− 1^) after application of the compound material, and ion content, physiological characteristics, and transcription of new cotton leaves at flowering and boll-forming stage were analyzed. The results showed that compared with saline stress, alkaline stress increased the contents of Na^+^, K^+^, SOD, and MDA in leaves. The application of the compound material reduced the content of Na^+^ but increased the K^+^/Na^+^ ratio, the activities of SOD, POD, and CAT, and REC. Transcriptome analysis revealed that after the application of the compound material, the Na^+^/H^+^ exchanger gene in cotton leaves was down-regulated, while the K^+^ transporter, K^+^ channel, and POD genes were up-regulated. Besides, the down-regulation of genes related to lignin synthesis in phenylalanine biosynthesis pathway had a close relationship with the ion content and physiological characteristics in leaves. The quantitative analysis with PCR proved the reliability of the results of RNA sequencing.

**Conclusion:**

These findings suggest that the compound material alleviated saline stress and alkaline stress on cotton leaves by regulating candidate genes in key biological pathways, which improves our understanding of the molecular mechanism of the compound material regulating the responses of cotton to saline stress and alkaline stress.

## Background

Soil salinization and alkalization are the main environmental factors that limit crop growth and yield [1]. Salinized soil and alkalized soils are widely distributed in arid and semi-arid regions around the world [2, 3], and the stresses caused by salinized and alkalized soils directly affect the ion balance of plant cells [4], which in turn affects physiological homeostasis [5, 6]. Many studies have shown that saline stress and alkaline stress are two different types of stress for plants [7], and the effect of alkaline stress on plants is more severe than that of saline stress [8]. Saline stress is mainly caused by neutral salt, while alkaline stress is mainly caused by alkaline salt [9]; saline stress generally causes ionic damage and osmotic stress in plants [10], and alkaline stress not only causes the above-mentioned damage to plants, but also increases the pH in plants [11]. Previous studies have found that the application of exogenous materials is one of the effective ways to regulate the responses of crops to saline stress and alkaline stress [12, 13]. Chemical modification can be used to regulate the responses of crops to saline stress and alkaline stress through replacing Na^+^ in soil by applying inorganic salts (e.g. calcium, aluminum sulfate, ferrous sulfate, etc.), or organic compounds (e.g. lignosulfonate, polyacrylamide, etc.), thus reducing soil salinity and alkalinity, promoting plant growth, and improving crop quality. Gong, et al. [12] found that melatonin regulated the enzyme activity and biosynthesis of polyamines and improved the tolerance of plants to alkaline stress. Faghih, et al. [13] showed that spraying salicylic acid and methyl jasmonate on the leaves could improve the defense system and antioxidant capacity of strawberry under salt stress. Therefore, it is of great importance to study the effects of the application of exogenous materials on the responses of plants to saline stress and alkaline stress.

Cotton (*Gossypium* spp.) is one of the most important economic crops in the world, among which *Gossypium hirsutum* L. has been widely planted and its planting area accounts for more than 95% of the global planting area. Although cotton is salt tolerant, its growth is affected by saline stress and alkaline stress [14]. According to reports, saline stress and alkaline stress affect seed germination, seedling growth, root growth, flowering, and boll number of cottons, resulting in a loss of yield [15–17]. Facing an increasing global demand for cotton, studies on regulating the damages caused by saline stress and alkaline stress on cotton have gained momentum [18]. Several genes regulating the response to saline and alkaline stress in cotton have been discovered. For example, ion channels and transporters can mitigate Na^+^ toxicity and K^+^/Na^+^ ratio homeostasis, and overexpression of NHX1 or SOS1 in cotton can improve salt tolerance [19]. *GhSOS3* and *GhCBL10* are involved in regulating the responses to saline stress and alkaline stress, and the *GhSOS3/GhCBL10-SOS2* network also plays a central role in *G. hirsutum* responses to saline stress and alkaline stress [20]. Besides, overexpression of the *OSCU/Zn-SOD* gene can improve the detoxification capacity of reactive oxygen species and improve the salt tolerance [21]. However, most of the previous studies were conducted through pot experiments or indoor culture experiments, and few was conducted through field experiments. Field experiments make the growth of crops very close to their natural growth, which can truly reflect the growth law of crops.

In this study, RNA-seq was used to analyze the transcriptional changes of cotton leaves under saline stress and alkaline stress, and to elucidate the molecular effects of the compound material on the improving saline and alkaline tolerance. We analyzed many genes related to plant antioxidant defense, K^+^/Na^+^ ratio transport, and lignin biosynthesis, and these genes may be involved in the regulation of the responses of cotton to saline stress and alkaline stress by the compound material. The main purposes of this experiment are: (1) to determine the differences in the responses of cotton to saline stress and alkaline stress; (2) to determine the differences in the effects of the compound material on K^+^, Na^+^, and physiological characteristics of cotton leaves; and (3) to provide insights on the relevant genes in the process of the regulation of the responses of cotton to saline stress and alkaline stress by the compound material.

## Results

### K^+^, Na^+^, and physiological characteristics of cotton leaves

**Fig. 1:**
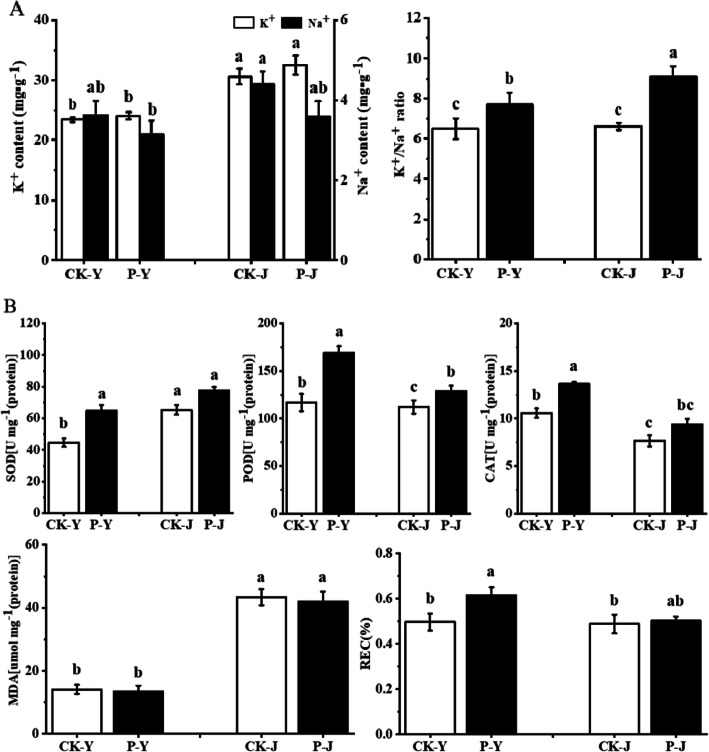
Effect of the application of compound material on K^+^ and Na^+^ contents and K^+^/Na^+^ ratio (**a**), antioxidative enzymes activity, and MDA and REC contents (**b**) in leaves

The K^+^ and Na^+^ contents of leaves for the Na_2_CO_3_ treatments (CK-J and P-J treatments) were higher than those for the NaCl treatments (CK-Y and P-Y treatments) (Fig. 1a). The contents of K^+^ and Na^+^ for the CK-J treatment were increased by 30.54% (*P < 0.05*) and 21.20% (*P < 0.05*), respectively compared with those for the CK-Y treatment (Fig. 1a). The K^+^/Na^+^ ratio for the P-Y and P-J treatments were increased (*P < 0.05*) and the Na^+^ contents were decreased (*P > 0.05*) after the application of compound material compared with those for the controls (CK-Y and CK-J treatments). For the P-Y treatment, there was no significant difference in the K^+^ content; the Na^+^ content was decreased (*P > 0.05*), and the K^+^/Na^+^ ratio was increased (*P > 0.05*), compared with those for the CK-Y treatment (Fig. 1a). For the P-J treatment, there was no significant difference in the K^+^ content; the Na^+^ content was decreased by 18.26% (*P > 0.05*), and the K^+^/Na^+^ ratio was increased by 37.11% (*P < 0.05*) compared with those for the CK-J treatment (Fig. 1a). Meanwhile, the K^+^ and Na^+^ contents and the K^+^/Na^+^ ratio for the P-J treatment were increased by 35.14% (*P < 0.05*), 14.11% (*P > 0.05*), and 18.27% (*P < 0.05*), respectively compared with those for the P-Y treatment (Fig. 1a).

The SOD activity for the CK-J treatment was increased by 46.29% (*P < 0.05*), while the POD and CAT activities were decreased by 4.09% (*P < 0.05*) and 27.60% (*P < 0.05*), respectively compared with those for the CK-Y treatment (Fig. 1b). The antioxidant enzyme activity for the P-Y and P-J treatments were increased compared with the controls (CK-Y and CK-J treatments) (Fig. 1b). The SOD, POD, and CAT activities and REC for the P-Y treatment were increased by 45.24, 44.71, 29.11, and 24.02%, respectively (*P < 0.05*), while there was no significant difference in the MDA content, compared with those for the CK-Y treatment. The POD and CAT activities for the P-J treatment were increased by 15.10% (*P < 0.05*) and 22.66% (*P > 0.05*), respectively, and there was no significant difference in the SOD activity and REC content, compared with those for the CK-J treatment (Fig. 1b). Meanwhile, there was no significant difference in the SOD activity and REC for the P-J treatment (*P > 0.05*), and the POD and CAT activities were decreased by 23.72% (*P < 0.05*) and 31.22% (*P < 0.05*), respectively, compared with those for the P-Y treatment (Fig. 1b).

### Overview of the Transcriptomic responses

Transcriptome of each sample was sequenced on Illumina paired-end sequencing platform. The number of reads generated ranged from 39 to 48 million, with a mean of 44 million reads for each sample. The reads were mapped onto the cotton reference transcriptome. The mapping ratio varied from 53.60 to 67.40%, with a mean of 64.11%. The counts of mapped reads were summarized at gene level (Additional file 1: Table S1, Additional file 2: Figure S1). The principal component analyses (PCA) was performed based on the gene counts (Additional file 3: Figure S2). The results showed that samples from NaCl and Na_2_CO_3_ treatments were clearly separated on the PC2 dimension, whereas the modified and unmodified samples were separated by at PC1 dimension. To verify the accuracy of RNA-seq data, six genes were randomly selected for quantitative RT-PCR (qRT-PCR) analysis. The expression abundances estimated by qRT-PCR and RNA-seq were highly correlated (R^2^ = 0.80, Additional file 4: Figure S3), indicating that the RNA-seq results were suitable for the subsequent analysis.

### Differentially expressed genes

**Fig. 2:**
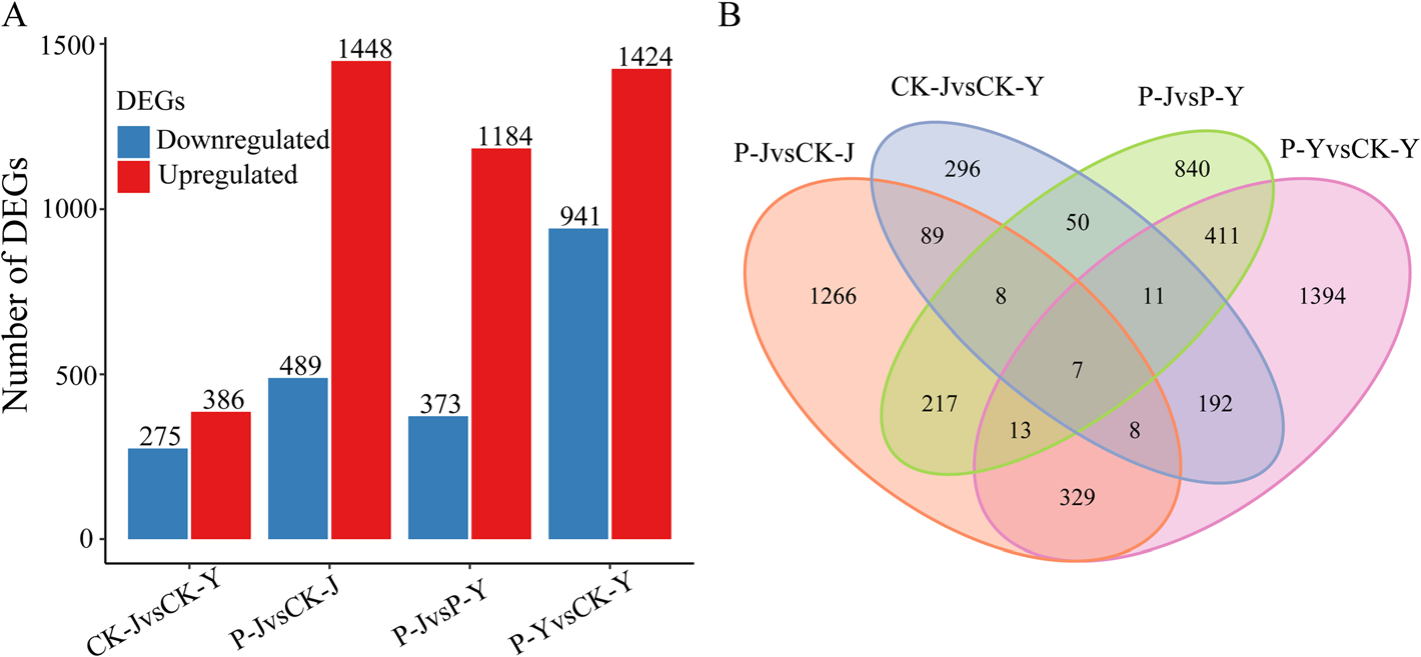
Transcriptome analysis of cotton leaves in response to the application of compound material regulating saline stress and alkaline stress. Numbers of DEGs identified in cotton leaves (**a**). Venn diagram of DEGs (**b**)

To determine the differences of transcriptional responses to the treatments, differentially expressed genes (DEGs) were identified by pair-wise comparisons of the samples. Compared with the CK-Y treatment, 386 genes were up-regulated and 275 genes were down-regulated for the CK-J treatment (Fig. 2a). A total of 1937 and 2365 DEGs were identified for the Na_2_CO_3_ treatments (CK-J and P-J treatments) and NaCl treatments (CK-Y and P-Y treatments), respectively (Fig. 2a). These results indicated that the expression patterns of more genes for NaCl treatments were altered compared with the Na_2_CO_3_ treatments. Compared with the CK-Y treatment, 1424 genes were up-regulated and 941 genes were down-regulated for the P-Y treatment. Compared with the CK-J treatment, 1448 genes were up-regulated and 489 genes were down-regulated for the P-J treatment (Fig. 2a). Compared with the P-Y treatment, 1184 genes for the P-J treatment were up-regulated and 373 genes were down-regulated. Venn diagram was draw to identify the common and specific DEGs. The results showed that there were 7 common differentially expressed genes for the four treatments (Fig. 2b).

### Enrichment analysis

**Fig. 3:**
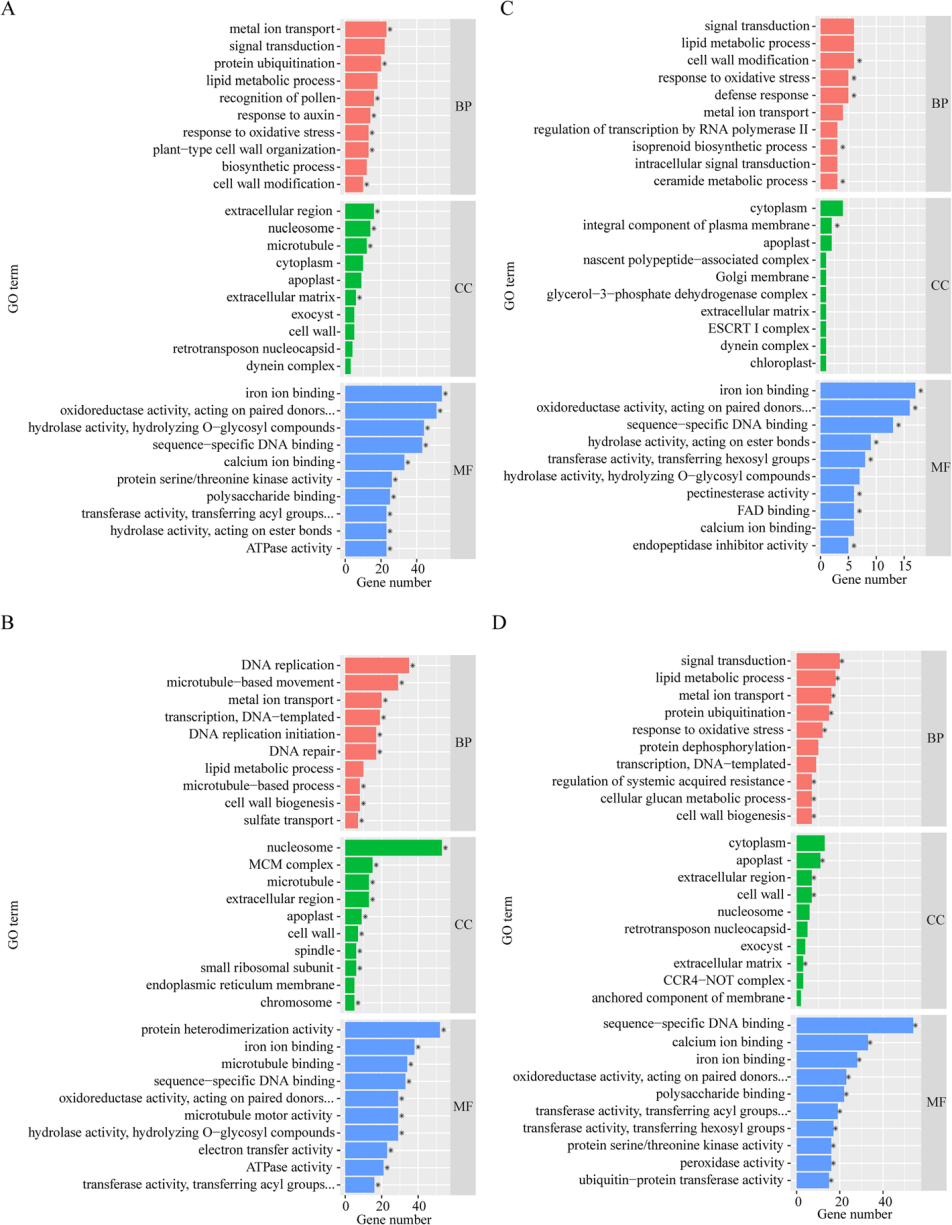
GO enrichment analysis of DEGs. The top 10 enriched GO terms in NaCl treatments (CK-Y and P-Y treatments). The top 10 enriched GO terms in Na_2_CO_3_ treatments (CK-J and P-J treatments) (**a**); The top 10 enriched GO terms in the controls (CK-J and CK-Y treatments) (**b**); The top 10 enriched GO terms in compound material treatments (P-J and P-Y treatments) (**c**); BP, CC, and MF represent biological process, cellular component, and molecular function, respectively (**d**). The asterisks represent the significant level of 0.05

GO enrichment analysis was performed on the DEGs identified in response to the compound material. For NaCl treatments (Fig. 3a), the top enriched GO terms for BP category were metal ion transport (GO:0030001), signal transduction (GO:0035556), and protein ubiquitination (GO:0016567), those for CC were extracellular region (GO:0005576), nucleosome (GO:0000786), and microtubule (GO:0005874), and those for MF were iron ion binding (GO:0005506), oxidoreductase activity (GO:0016705), and hydrolase activity (GO:0004553). For Na_2_CO_3_ treatments (Fig. 3b), the top enriched GO terms for BP were metal DNA replication (GO:0006260), microtubule−based movement (GO:0007018), and metal ion transport (GO:0030001), those for CC were nucleosome (GO:0000786), MCM complex (GO:0042555), and microtubule (GO:0005874), and those for MF were protein heterodimerization activity (GO:0046982), iron ion binding (GO:0005506), and microtubule binding (GO:0008017). For the controls (Fig. 3c), the top enriched GO terms for BP were signal transduction (GO:0007165), lipid metabolic process (GO:0006629), and cell wall modification (GO:0042545), those for CC were cytoplasm (GO:0005737), integral component of plasma membrane (GO:0005887), and apoplast (GO:0048046), and those for MF were iron ion binding (GO:0005506), oxidoreductase activity, acting on paired donors (GO:0016705), and sequence−specific DNA binding (GO:0043565). For compound material treatments (Fig. 3d), the top enriched GO terms for BP were signal transduction (GO:0007165), lipid metabolic process (GO:0006629), and metal ion transport (GO:0030001), those for CC were cytoplasm (GO:0005737), apoplast (GO:0048046), and extracellular region (GO:0005576), and those for MF were sequence−specific DNA binding (GO:0043565), calcium ion binding (GO:0005509), and iron ion binding (GO:0005506).

To further understand the molecular interactions among the DEGs, KEGG enrichment analysis was carried out. The results showed phenylpropanoid biosynthesis, pertussis, and brassinosteroid biosynthesis pathways were significantly enriched in NaCl treatments (CK-Y and P-Y treatments) (Additional file 5: Figure S4A); systemic lupus erythematosus and alcoholism pathways were significantly enriched in Na_2_CO_3_ treatments (CK-J and P-J treatments) (Additional file 5: Figure S4B); phenylpropanoid biosynthesis, glycerolipid metabolism, amino sugar and nucleotide sugar metabolism, and pentose and glucuronate interconversions pathways were significantly enriched in the controls (CK-J and CK-Y treatments) (Additional file 5: Figure S4C); phenylpropanoid biosynthesis and alpha-Linolenic acid metabolism pathways were significantly enriched in compound material treatments (P-J and P-Y treatments) (Additional file 5: Figure S4D).

### Response of the salt ion transporter in cotton leaves

The transporter-mediated salt ion balance in cotton leaves is an important part involved in the regulation of the responses of cotton to saline stress and alkaline stress by compound material. For NaCl treatments (CK-Y and P-Y treatments), K^+^ transporter and K^+^ channel genes were significantly regulated by the compound material, and four up-regulated K^+^ channel genes (GH_A13G1568, GH_D01G0882, GH_D13G1517, and GH_A01G0868) were identified. Besides, one K^+^ transporter gene (GH_D08G2294) was up-regulated, and one sodium/hydrogen exchanger gene (GH_A09G0801) was down-regulated (Table 1). For Na_2_CO_3_ treatments (CK-J and P-J treatments), the expression levels of genes related to K^+^ transporter (GH_D05G2808) and K^+^ channel (GH_A01G0868, GH_D01G0882, and GH_D13G1517) changed significantly, and all were up-regulated (Table 1). For compound material treatments (P-J and P-Y treatments), one K^+^ channel gene (GH_A05G1107) was down-regulated (Table 1).

**Table 1:** Expression patterns of DEGs involved in salt ions transport

	Gene ID	log2FC	*P* value	Padj	Description
CK-Y vs. P-Y	GH_A09G0801	−1.2	0.04993	0.83714	sodium/hydrogen exchanger 3
	GH_D08G2294	1.31	0.00057	0.15126	potassium transporter 1
	GH_A13G1568	2.49	0.00742	0.55377	two-pore potassium channel 1
	GH_D01G0882	2.67	0.01501	0.69433	two-pore potassium channel 1
	GH_D13G1517	2.85	0.00823	0.57328	two-pore potassium channel 1
	GH_A01G0868	2.99	0.00726	0.55086	two-pore potassium channel 1
CK-J vs. P-J	GH_A01G0868	2.20	0.04683	0.98810	two-pore potassium channel 1
	GH_D01G0882	2.35	0.03191	0.93991	two-pore potassium channel 1
	GH_D13G1517	2.55	0.01693	0.82443	two-pore potassium channel 1
	GH_D05G2808	2.03	0.00451	0.57112	potassium transporter 2
P-J vs. P-Y	GH_A05G1107	−1.20	0.00348	0.47248	potassium channel SKOR

### Regulation of antioxidative defense in cotton leaves

Many DEGs in cotton leaves were significantly enriched in oxidoreductase activity Go term. Eight peroxidase genes (GH_A06G1119, GH_D11G2319, GH_D10G1060, GH_A12G2651, GH_A05G0628, GH_D10G1977, GH_D06G1268, and GH_A06G1247) for NaCl treatments were up-regulated, and three peroxidase genes (GH_A05G4223, GH_A06G1247, and GH_A05G0628) for Na_2_CO_3_ treatments were up-regulated. Besides, one peroxidase gene (GH_A05G1582) for compound material treatments was down-regulated, and five peroxidase gene (GH_A03G1283, GH_D03G1634, GH_D04G0154, GH_D03G1633, and GH_D08G2611) was up-regulated. One peroxidase gene (GH_D05G1612) for the controls was down-regulated, and one peroxidase gene (GH_D10G1977) was up-regulated (Table 2).

**Table 2:** Expression patterns of DEGs involved in peroxidase

	Gene ID	log2FC	*P* value	Padj	Description
CK-Y vs. P-Y	GH_A06G1119	3.29	0.04162	0.81913	peroxidase A2
	GH_D11G2319	17.66	0.00005	0.02426	peroxidase A2
	GH_D10G1060	2.50	0.04006	0.81328	peroxidase 50
	GH_A12G2651	2.57	0.01132	0.63957	peroxidase 5
	GH_A05G0628	4.46	0.03935	0.81251	peroxidase 46
	GH_D10G1977	19.80	0.00000	0.00046	peroxidase 29
	GH_D06G1268	4.54	0.00327	0.39170	peroxidase 12
	GH_A06G1247	3.44	0.00624	0.52647	peroxidase 12
CK-J vs. P-J	GH_A05G4223	3.40	0.01666	0.82443	peroxidase P7
	GH_A06G1247	3.85	0.00266	0.47075	peroxidase 12
	GH_A05G0628	4.35	0.04211	0.97147	peroxidase 46
P-J vs. P-Y	GH_A05G1582	−2.30	0.04592	1.00000	peroxidase 19
	GH_A03G1283	1.42	0.03043	1.00000	peroxidase 3
	GH_D03G1634	1.76	0.02381	0.96048	peroxidase 4
	GH_D04G0154	2.85	0.01578	0.85903	peroxidase P7
	GH_D03G1633	5.85	0.01184	0.78791	peroxidase 4
	GH_D08G2611	8.65	0.01451	0.84244	peroxidase 53
CK-J vs. CK-Y	GH_D05G1612	−1.74	0.01021	1.00000	peroxidase 19
	GH_D10G1977	17.04	0.00002	0.02101	peroxidase 29

### Analysis of correlation between transcription genes of K^+^, Na^+^, and physiological characteristics

**Fig. 4:**
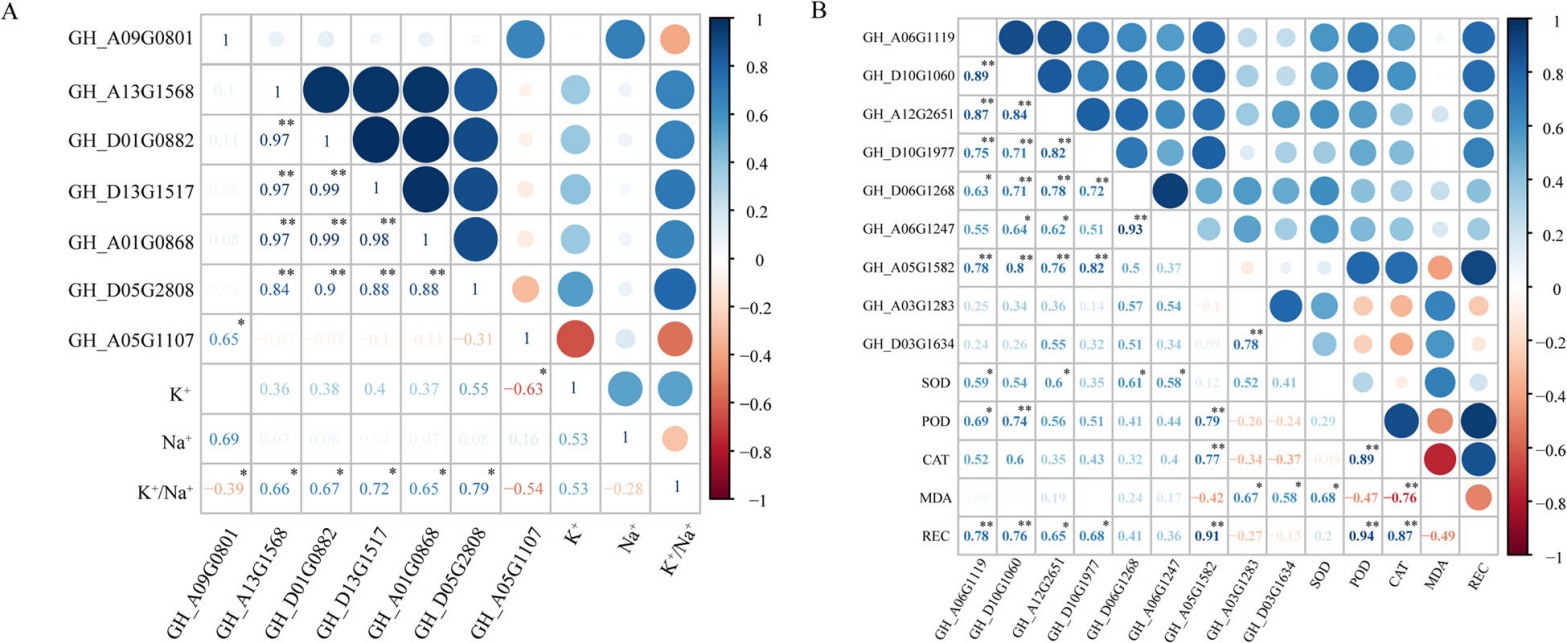
Correlation analysis between transcription genes of K^+^, Na^+^ (**a**) and physiological characteristics (**b**)

The correlation coefficients (r) between transcription genes of K^+^, Na^+^, and physiological characteristics and results of significance tests are shown in Fig. 4. GH_A09G0801 was positively correlated with Na^+^ content (*P < 0.05*); GH_A13G1568, GH_D01G0882, GH_D13G1517, GH_A01G0868, and GH_D05G2808 were positively correlated with K^+^/Na^+^ ratio (*P < 0.05*) (Fig. 4a). GH_A06G1119, GH_D10G1060, GH_A12G2651, GH_D10G1977, GH_D06G1268, and GH_A06G1247 were positively correlated with SOD activity (*P < 0.05*); GH_A06G1119 (*P < 0.05*), GH_D10G1060 (*P < 0.01*), and GH_A05G1582 (*P < 0.01*) were positively correlated with POD activity; GH_D10G1060 (*P < 0.05*) and GH_A05G1582 (*P < 0.01*) were positively correlated with CAT activity; GH_A03G1283 and GH_D03G1634 were positively correlated with MDA content (*P < 0.01*); GH_A12G2651 (*P < 0.05*), GH_D10G1977 (*P < 0.05*), GH_A06G1119 (*P < 0.01*), GH_D10G1060 (*P < 0.01*), and GH_A05G1582 (*P < 0.01*) were positively correlated with REC (Fig. 4b).

### DEGs involved in the phenylpropanoid biosynthesis pathway

**Fig. 5:**
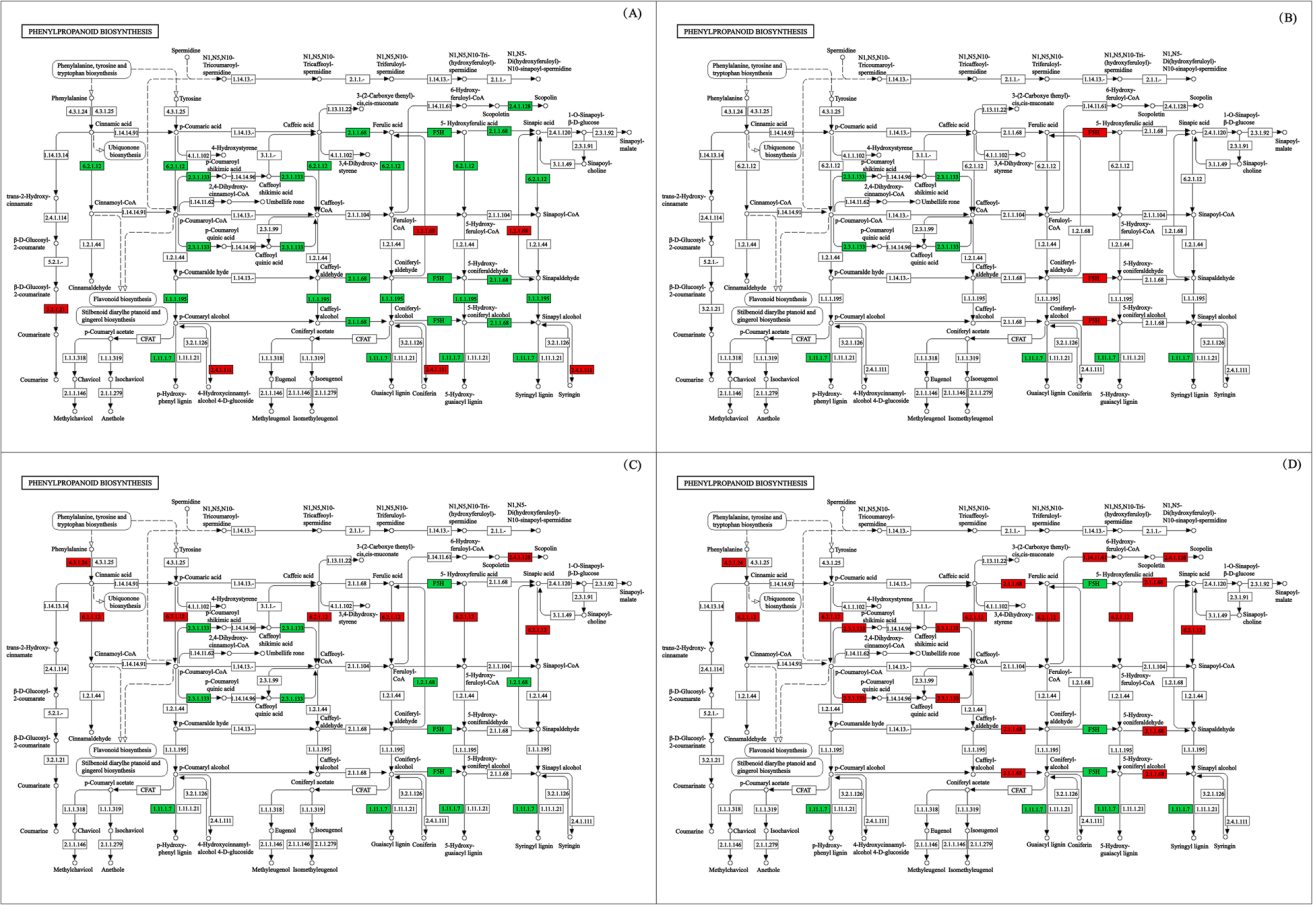
Representation of genes related to phenylpropanoid biosynthesis pathway (https://www.kegg.jp/dbget-bin/www_bget?map00940). The red frames represent up-regulated DEGs, the green frames represent down-regulated DEGs. Pathway in NaCl treatments (CK-Y and P-Y treatments (**a**); Pathway in Na_2_CO_3_ treatments (CK-J and P-J treatments) (**b**); Pathway in control treatments (CK-J and CK-Y treatments) (**c**); Pathway in compound material treatments (P-J and P-Y treatments) (**d**) (State: We obtained the appropriate copyright permission to modify the phenylpropanoid biosynthesis pathway)

The expression of the genes involved in phenylpropanoid biosynthesis (https://www.kegg.jp/dbget-bin/www_bget?map00940) of cotton leaves in response to the application of the compound material was analyzed (Fig. 5). For NaCl treatments, the DEGs involved in beta-glucosidase (EC:3.2.1.21), coniferyl-alcohol glucosyltransferase (EC:2.4.1.111), and coniferyl-aldehyde dehydrogenase (EC:1.2.1.68) were up-regulated, while DEGs involved in scopoletin glucosyltransferase (EC:2.4.1.128), caffeic acid 3-O-methyltransferase (EC:2.1.1.68), ferulate-5-hydroxylase (EC:1.14.-.-), 4-coumarate--CoA ligase (EC:6.2.1.12), shikimate O-hydroxycinnamoyl transferase (EC:2.3.1.133), cinnamyl-alcohol dehydrogenase (EC:1.1.1.195), and peroxidase (EC:1.11.1.7) were down-regulated (Fig. 5a). For Na_2_CO_3_ treatments, the DEGs involved in ferulate-5-hydroxylase (EC:1.14.-.-) were up-regulated, while DEGs involved in shikimate O-hydroxycinnamoyl transferase (EC:2.3.1.133) and peroxidase (EC:1.11.1.7) were down-regulated (Fig. 5b). For the controls, the DEGs involved in phenylalanine ammonia-lyase (EC:4.3.1.24), scopoletin glucosyltransferase (EC:2.4.1.128), and 4-coumarate--CoA ligase (EC:6.2.1.12) were up-regulated, while DEGs involved in shikimate O-hydroxycinnamoyl transferase (EC:2.3.1.133), ferulate-5-hydroxylase (EC:1.14.-.-), coniferyl-aldehyde dehydrogenase (EC:1.2.1.68), and peroxidase (EC:1.11.1.7) were down-regulated (Fig. 5c). For compound material treatments, the DEGs involved in phenylalanine ammonia-lyase (EC:4.3.1.24), feruloyl-CoA 6-hydroxylase (EC:1.14.11.61), scopoletin glucosyltransferase (EC:2.4.1.128), caffeic acid 3-O-methyltransferase (EC:2.1.1.68), 4-coumarate--CoA ligase (EC:6.2.1.12), shikimate O-hydroxycinnamoyl transferase (EC:2.3.1.133), and caffeic acid 3-O-methyltransferase (EC:2.1.1.68) were up-regulated, while DEGs involved in ferulate-5-hydroxylase (EC:1.14.-.-) and peroxidase (EC:1.11.1.7) were down-regulated (Fig. 5d).

## Discussion

Under saline stress and alkaline stress, excessive Na^+^ will accumulate in plant leaves, inhibiting the transport of K^+^ and causing K^+^ and Na^+^ ion imbalance in plant cells [22]. However, the regulations of ion balance are different under saline stress and alkaline stress. Wang, et al. [9] showed that the Na^+^ content under alkaline stress was greater than that under saline stress in pot experiments. In this study, the Na^+^ and K^+^ contents of cotton leaves under alkaline stress were significantly higher than those under saline stress, and there was no significant difference in K^+^/Na^+^ ratio. In order to regulate the responses of cotton to saline stress and alkaline stress, the compound material was applied in field experiments. Zhang, et al. [23] found that the Na^+^/H^+^ exchanger 4 of sesame aerial parts was up-regulated under saline stress through hydroponic culture. Zhao, et al. [24] found 17 Na^+^/H^+^ antiporters in the root of chrysanthemum in response to saline stress. Niu, et al. [25] found that salinity significantly decreased the expression of Na^+^/H^+^ exchanger 4 in leaf veins. In the study, for compound material treatments, the Na^+^ content was decreased; this might be because the stress signal of Na^+^ could be quickly inhibited by the down-regulation of a Na^+^/H^+^ exchanger 3 gene when the compound material was applied to salinized soil. Moreover, Na^+^/H^+^ exchangers reduced the accumulation of Na^+^ by fixing Na^+^ and storing it in vacuoles [26, 27]. Huang, et al. [28] found that the potassium channel KAT1 of the aboveground part of barley was down-regulated under saline stress. In this study, under saline stress and alkaline stress, the K^+^ content and K^+^/Na^+^ ratio for compound material treatments were increased; this might be because the application of compound material increased the transcription level of certain genes encoding K transporters and K channels in cotton leaves. In particular, under saline stress, several genes of two-pore K^+^ channel 1 and K^+^ transporter 1 for saline treatments were up-regulated, and several genes of K^+^ transporter 2 and two-pore K^+^ channel 1 for alkaline treatments were also up-regulated. Among them, the up-regulation of GH_A13G1568, GH_D01G0882, GH_D13G1517, GH_A01G0868, and GH_D05G2808 genes had positive effects on K^+^/Na^+^ ratio, which suggested that the compound material could alleviate saline stress and alkaline stress by regulating ion balance in leaves. Moreover, a K^+^ channel SKOR gene was down-regulated for the Na_2_CO_3_ treatments compared with that for the NaCl treatments, suggesting that the application of the compound material had a better effect on the recovery of K^+^ content of cottons under saline stress.

The differences in K^+^ and Na^+^ contents between NaCl treatments and Na_2_CO_3_ treatments were due to the different physiological damages suffered by cotton. In this study, it was found that alkaline stress caused more physiological damage to cotton leaves than saline stress. Gong, et al. [12] found that the application of exogenous substances could promote the antioxidant system to remove excess free radicals and regulate physiological damage. In this study, we also found that the application of the compound material could regulate the physiological damage suffered by cotton. It had the same effect on the regulation of antioxidant enzymes under saline stress and alkaline stress, but the degrees of the effects were different. For example, no matter under saline stress or under alkaline stress, the compound material could increase the SOD, POD, and CAT activities of cotton leaves. This might be because a large number of DEGs were related to oxidoreductase activity for CK-Y, P-Y, CK-J, and P-J treatments. A total of 51 DEGs of oxidoreductase activity Go term for NaCl treatments were regulated, and 29 DEGs for Na_2_CO_3_ treatments were regulated; moreover, the compound material activated the oxidative stress response under saline stress and alkaline stress. Among them, the compound material significantly increased the activities of SOD and CAT under saline stress. This might be because the up-regulation of GH_A06G1119, GH_A12G2651, GH_D06G1268, and GH_A06G1247 increased SOD activity, while the up-regulation of GH_D10G1060 increased CAT activity; however, the compound material did not significantly increase the activities of SOD and CAT under alkaline stress, indicating that the compound material had less effect on the activities of SOD and CAT under alkaline stress, and SOD activity might not play a role in saline and alkaline tolerances of cotton [29]. Studies have shown that POD is the main detoxification enzyme of plants under saline stress and alkaline stress [30]. This study found that the compound material significantly increased the POD activity of cotton leaves under saline stress and alkaline stress. This might be because the genes related to peroxidase for CK-Y, P-Y, CK-J, and P-J treatments were up-regulated, and the expression of antioxidant enzyme genes was also up-regulated, leading to the improvement of the tolerance of cotton to saline stress and alkaline stress after applying the compound material; besides, the application of compound material under saline stress up-regulated, the expression of a great number of antioxidant enzyme genes. Luo, et al. [31] showed that *SOD1* and *CAT1* genes were involved in the cottons’ response to saline stress. Geng, et al. [32] found that the *POD7* and *SOD [Cu-Zn]* genes of the salt-tolerant varieties of sugar beet were significantly up-regulated. However, in our study, the application of compound material only significantly regulated the POD *A2/50/5/46/29/12/P7* in cotton leaves. This might be because the permeability of the soil in the field enhanced root vitality and promoted cotton’s tolerance, so only peroxidase-related genes were involved in the responses to saline stress and alkaline stress. We also noticed that under saline stress and alkaline stress, the REC and MDA in leaves were affected. The difference in REC under saline stress and alkaline stress was not significant, but the MDA content under alkaline stress was much higher than that under saline stress. Cui, et al. [33] found that the RCE of peanut leaves was increased under saline stress. Gong, et al. [12] found that the leaf MDA content of *Malus hupehensis* Rehd. under alkaline stress was decreased by applying melatonin. Our study found that the application of the compound material to cottons under saline stress and alkaline stress increased the REC content of cotton leaves. Among them, only the increase in REC of cottons under saline stress was significant. This might be because the up-regulation of GH_A06G1119, GH_D10G1060, GH_A12G2651, and GH_D10G1977 increased the REC content, indicating that the compound material decreased the effects of saline stress and alkaline stress on the stability and integrity of the cell membrane. Moreover, the effect of the compound material on the cell membrane of cottons under saline stress was more significant than that under alkaline stress.

The genes for lignin biosynthesis are dynamically regulated at different levels to protect plant cell metabolism from oxidative damage [34]. In the transcription of data, functional analysis of DEGs was performed through KEGG and GO enrichment analysis, and it was found that a large number of genes were involved in the phenylpropanoid biosynthesis pathway. The phenylpropanoid biosynthesis pathway is one of the most important secondary metabolite pathways in plants, and is related to the plant’s response to saline stress and alkaline stress [35]. The lignin metabolites produced in this pathway are of great significance for plants to resist abiotic stress [35, 36]. Besides, four lignins (p-hydroxyphenyl lignin, guaiacyl lignin, 5-hydroxy-guaiacyl lignin, and syringyl lignin) were aggregated by four monomers (p-coumaryl alcohol, coniferyl alcohol, 5-hydroxy-coniferyl alcohol, and sinapyl alcohol), while four alcohols were catalyzed by peroxidase (EC: 1.11.1.7), leading to the formation of these lignins (Fig. 5). Shen, et al. [37] found that seven genes related to lignin biosynthesis in *Arabidopsis thaliana* were up-regulated under saline stress. We found that 4CL, HCT, COMT, TOGT1, F5H, CAD, and POD enzymes for the P-Y treatment were down-regulated compared with those for the CK-Y treatment, suggesting that these enzymes might play a role in the decrease of lignin synthesis and the protection of cotton from the damage caused by saline stress by compound material. Moreover, previous studies have found that 4CL enzyme changes the accumulation of lignin [38], HCT enzyme modifies H lignin (p-hydroxyphenyl lignin) [39], COMT enzyme participates in the biosynthesis of S lignin (syringyl lignin) [40], F5H enzyme regulates the composition of S/G lignin (syringyl (S)/guaiacyl (G) lignin) [41], CAD enzymes change the lignin content and structure [42], and POD enzymes participate in lignin biosynthesis and affect plant growth [43]. In this study, the expression levels of HCT and POD enzymes for the P-J treatment were down-regulated compared with those for the CK-J treatment, suggesting that 5-O-Caffeoylshikimic acid and caffeoyl quinic acid could not be converted into caffeoyl-CoA. However, caffeoyl-CoA is an essential intermediate for lignin biosynthesis [44]. The above indicates that under both saline stress and alkaline stress, the application of compound material down-regulates the peroxidase (EC: 1.11.1.7), which might be because the compound material reduces the lignin biosynthesis under saline stress and alkaline stress.

## Conclusions

**Fig. 6:**
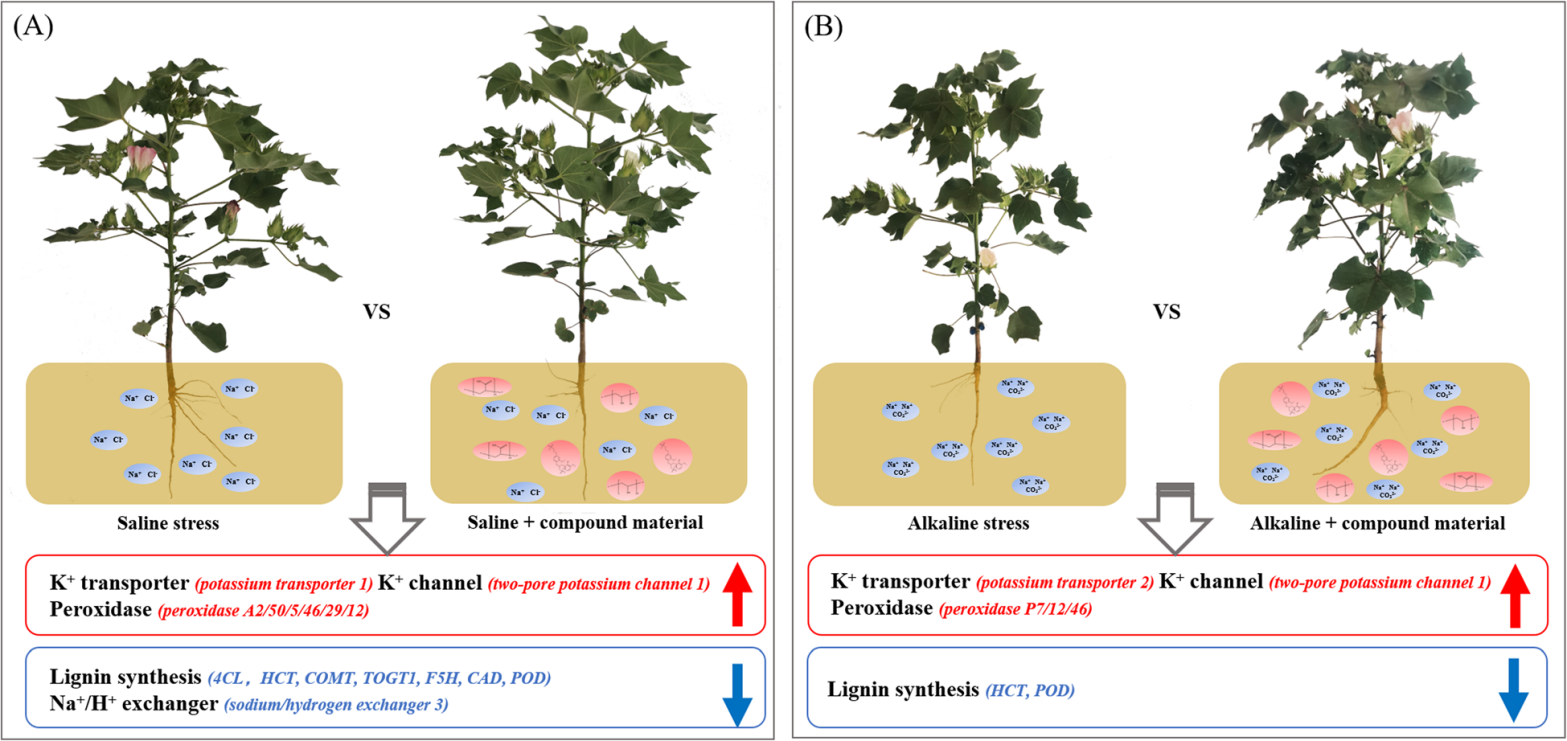
Proposed model for the function of compound material in regulating saline stress (**a**) and alkaline stress (**b**) of cotton leaves. The up-pointing red arrows mean that the candidate genes are up-regulated; the down-pointing blue arrows mean that the candidate genes are down-regulated

Field test results showed that saline stress and alkaline stress were two different stresses. Under saline stress, the contents of Na^+^ and MDA in cotton leaves were high, the activities of POD and CAT were low, and the effect of alkaline stress were greater than that of saline stress. The application of the compound material was mainly to increase cotton K^+^/Na^+^ ratio and POD activity to increase saline and alkaline tolerance of cotton. Through transcriptome analysis, it was further found that K^+^ transporter genes and peroxidase-related genes were up-regulated during the regulation of the responses of cotton to saline stress and alkaline stress by the compound material; and the enzymes involved in lignin biosynthesis were down-regulated, which protected cotton from the damage caused by saline stress and alkaline stress (Fig. 6). Among them, these up-regulated genes and down-regulated enzymes were abundant in cotton leaves during the regulation of the responses of cotton to saline stress by compound material. Moreover, these differentially expressed genes obtained in field trials have high stability, which is applicable in the field breeding in the future.

## Methods

### Experiment site

Cotton (Xinluzao 62) seeds were obtained from Cotton Crops Research Institute (Shihezi City, Xinjiang, China). This experiment was conducted at the Experimental Station of Grape Research Institute in Shihezi City, Xinjiang Province, China (44°20′ N、86°03′ E). The soil is a desert grey soil. Soil basic characteristics are shown in Table 3.

**Table 3:** Soil basic characteristics of the tested soil [45]

Item	Value
pH	7.72
cation exchange capacity (CEC)	17.32 coml kg^−1^
Organic matter contents	12.5 g kg^− 1^
Alkali-hydrolyzable nitrogen	54 mg kg ^− 1^
Available phosphorus	11.7 mg kg ^− 1^
Available potassium	218 mg kg^− 1^

### Experimental materials and experimental design

This experiment was conducted from April 20th to September 20th, 2018. The cotton (variety Xinluzao 62) and a compound material were used as the experimental material in this study. Compound material was a mixture of calcium lignosulfonate, manganese sulfate, zinc sulfate, ferric sulfate, and boric acid, anionic polyacrylamide, polyvinyl alcohol (Mass ratio: 4:4:4:2:1:0.5:0.5).

This experiment employed a randomized block design. There were four treatments in total and each treatment had three repetitions: (1) P-Y treatment (compound material of 300 kg hm^− 2^ was applied and NaCl of 8 g kg^− 1^ was mixed fully with the plough layer), (2) P-J treatment (compound material of 300 kg hm^− 2^ was applied and Na_2_CO_3_ of 8 g kg^− 1^ was mixed fully with the plough layer), (3) CK-Y treatment (no compound material was applied and NaCl of 8 g kg^− 1^ was mixed fully with the plough layer), and (4) CK-J treatment (no compound material was applied and Na_2_CO_3_ of 8 g kg^− 1^ was mixed fully with the plough layer). On April 20th, 2018, soils were put into plastic barrels (0.5 m in diameter and 0.6 m in height) keeping the status of soil layer, and then barrels were buried back to the field. After that, NaCl and Na_2_CO_3_ were applied. The pH and EC of salinized soil were 8.24 and 1.84 s m^− 1^, respectively, and those of alkalized soil were 9.78 and 1.03 s m^− 1^, respectively. On April 29th, for all treatments, 0.2 g kg^− 2^ urea and 0.4 g kg^− 2^ synthetic fertilizer (formulated for drip irrigation; N: 0.07 g kg^− 2^; P: 0.07 g kg^− 2^; K: 0.07 g kg^− 2^) were applied. On May 4th, cotton was sown; and after emergence, six seedlings were retained in each barrel. On May 6th, the compound material was applied after diluting with water. Both the fertilizer and compound material were applied to soils with drip irrigation at once. The seedlings were irrigated for the first time on June 25th. The irrigation cycle was 3 days. At the flowering and boll-forming stage (August 19th), new leaves were collected for transcriptome sequencing (three replicates per treatment). All samples were immediately placed in liquid nitrogen and stored at − 80 °C until use.

### Plant physiological analysis

The activities of antioxidant enzymes were assayed in leaves (0.5 g) using spectrophotometric methods [46–48]. Superoxide dismutase (SOD) activity was measured (560 nm) based on the NBT photochemical reduction [46]. The peroxidase (POD) activity was measured (470 nm) based on the absorbance caused by guaiacol [47]. The catalase (CAT) activity was measured (240 nm) based on the reaction of potassium phosphate buffer and H_2_O_2_ [48].

Malondialdehyde (MDA) was measured in terms of a thiobarbituric acid reactive substances (TBARS) content of the leaf samples (nmol/g; extinction coefficient: 155 mM cm^− 1^) [49]. For relative electrical conductivity (REC) [50], 0.1 g of fresh leaves were cut into 1 cm slices, placed in 10 mL of deionized water, and shaken for 24 h at room temperature on a rotary shaker (QL200H, Shanghai, China). Then, electrical conductivity of the solution (L1) was measured using a conductivity meter (EM38, ICT international, Armidale, NSW, Australia). The solution was boiled for 15 min and cooled to room temperature, and electrical conductivity (L2) was again measured. Finally, REC was calculated (REC = L1/L2).

The Na^+^ and K^+^ contents of the leaf samples were determined according to the method of Bao [45]. Leaf samples were immersed in 98% H_2_SO_4_ and 30% H_2_O_2_, and a flame spectrophotometer (AP1200 type, Shanghai, China) was used for the determination.

### Transcriptome sequencing and data analysis

In this study, total RNA of 12 samples was extracted [51], PolyA mRNA in total RNA was enriched by Oligo (dT) magnetic beads, and RNA was interrupted about 300 bp in length by ion interruption. The first strand of cDNA was synthesized using 6 base random primers and reverse transcriptase as template, and the second strand cDNA was synthesized using the first strand cDNA as template. After the construction of the library, the library fragments were enriched by PCR amplification, and then the library was selected according to the size of the fragments (450 bp). Then, Agilent 2100 Bioanalyzer (Agilent Technologies, Palo Alto, Calif.) was used to check the total concentration and effective concentration of the library. Then, according to the effective concentration of the library and the amount of data needed by the library, the libraries containing different Index sequences are mixed proportionally. After RNA extraction, purification and library construction, these samples were sequenced by Next-Generation Sequencing (NGS) based on Illumina Sequencing platform [51]. The RNA library construction was carried out by Shanghai Personal Bioinformatics Co., Ltd. (http://www.personalbio.cn/).

The quality of the reads was checked using FastQC (http://www.bioinformatics.babraham.ac.uk/projects/fastqc/). Fastp was used to remove the adapter and low-quality sequences in the reads [52]. Cotton genome sequence of (*Gossypium hirsutum,* ZJU) were downloaded from Hu, et al. [53] and used as the reference genome (https://www.cottongen.org/species/Gossypium_hirsutum/ZJU-AD1_v2.1). The clean reads were qausi-mapped on to all annotated transcripts using Salmon [54]. Expression abundance at the unit of transcript per million (TPM) was calculated at gene level. DESeq2 was used to identify the differentially expressed genes (DEGs) between samples with the thresholds of adjusted *p*-value less than 1 and absolute value of log2(fold change) larger than 1 [55]. Principal component analysis (PCA) was performed to display the transcriptomic similarity among the samples based on the counts of top 1000 genes. Gene ontology (GO) and Kyoto Encyclopedia of Genes and Genomes (KEGG) enrichment analyses were conducted using clusterProfiler [56]. Pathway analysis done using the KEGG mapping method. The Unigene sequences were mapped to the KEGG biochemical pathways according to the EC distribution in the pathway database [57, 58].

### Quantitative real-time PCR validation

To validate the RNA-seq data, six DEGs from the pathway enrichment analysis were selected for qRT-PCR analysis. Samples of RNA-Seq were reverse transcribed into cDNA for real-time qPCR validation using the PrimeScript™ 1st stand cDNA Synthesis Kit and SYBR Green Master Mixes (Vazyme Biotech, Nanjing, China). qRT-PCR was performed on a fluorescence quantitative system TIB8600 (Taipu, Biotech, Xiamen, China). Each sample was measured with three biological and three technical replicates, and the relative expression levels were calculated using the 2^-⊿⊿Ct^ method. The endogenous reference gene used was GhEF1α. The gene-specific primers are listed in Table S2 (Additional file 1).

### Statistical analysis

One-way analysis of variance (ANOVA) was performed for K^+^ and Na^+^ contents and physiological characteristics of cotton leaves (Duncan test, *P* < 0.05, SPSS 22.0). All the above analyses were performed in R software (Version 3.2.3, http://www.r-project.org) using the Vegan and Origin 8.0 software.

## Supplementary information

**Additional file 1: Table S1.** Statistical analyses and mapping results of RNA sequencing reads. **Table S2.** Primers used in qRT-PCR analysis.

**Additional file 2: Figure S1.** An overview of RNA-Seq data

**Additional file 3: Figure S2.** PCA clustering based on RNA-Seq data

**Additional file 4: Figure S3.** Correlation analysis between qRT-PCR and RNA-Seq data based on log2 fold change

**Additional file 5: Figure S4.** KEGG enrichment analysis of DEGs. (A) Pathways in NaCl treatments (CK-Y and P-Y treatments). (B) Pathways in Na_2_CO_3_ treatments (CK-J and P-J treatments). (C) Pathways in the controls (CK-J and CK-Y treatments). (D) Pathways in compound material treatments (P-J and P-Y treatments).

## Data Availability

The raw RNA-seq data are available from NCBI Sequence Read Archive (SRA) database under accession PRJNA660498 (https://www.ncbi.nlm.nih.gov/sra/PRJNA660498). All data generated or analyzed during this study are available from the corresponding author on reasonable request.

## Abbreviations

SOD: Superoxide dismutase
POD: Peroxidase
CAT: Catalase
MDA: Malondialdehyde
REC: Relative electrical conductivity
Go: Gene ontology
KEGG: Kyoto Encyclopedia of Genes and Genomes
DEGs: Differentially expressed genes (DEGs)
