# Supplementary figures and images for: Application of compound material alleviates saline and alkaline stress in cotton leaves through regulation of the transcriptome
Source: BMC Plant Biol. 2020 Oct 8;20:462. doi: 10.1186/s12870-020-02649-0 (PMC7542905; doi:10.1186/s12870-020-02649-0)

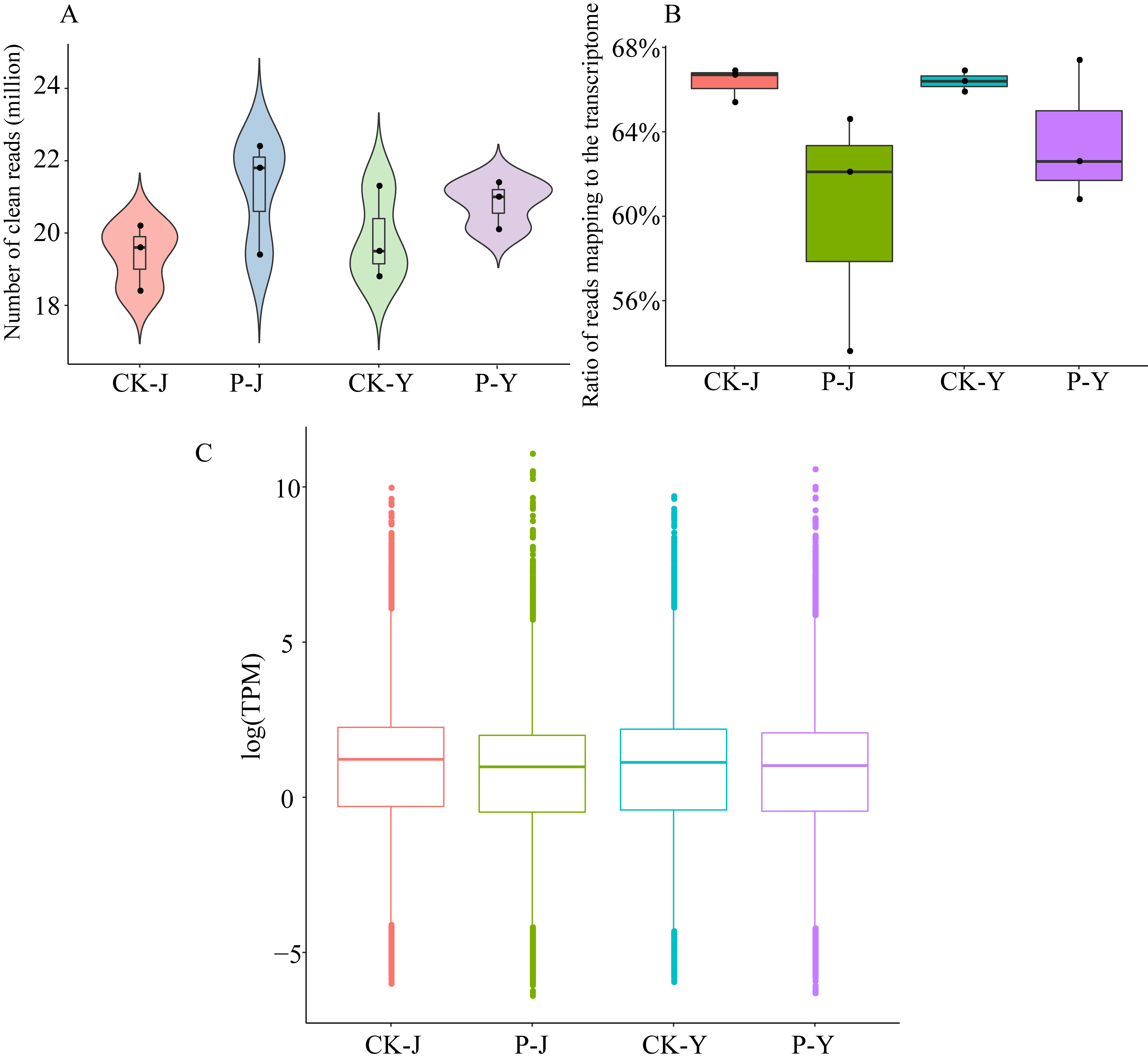

Supplement: Supplementary file 2 — Additional file 2: Figure S1. An overview of RNA-Seq data [file 12870_2020_2649_MOESM2_ESM.tif]

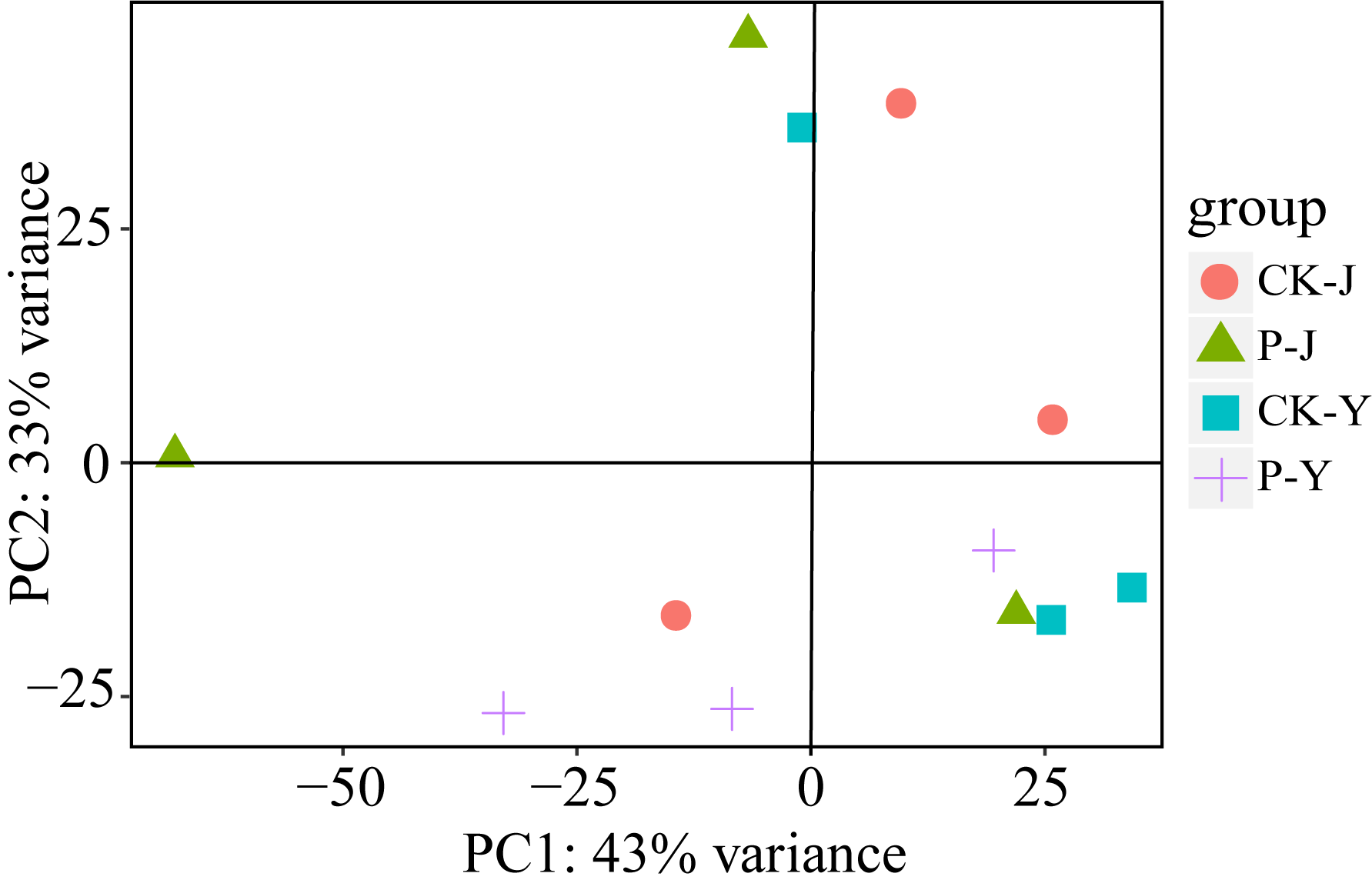

Supplement: Supplementary file 3 — Additional file 3: Figure S2. PCA clustering based on RNA-Seq data [file 12870_2020_2649_MOESM3_ESM.tif]
